# Fecal Malodor Detection Using Low-Cost Electrochemical Sensors

**DOI:** 10.3390/s20102888

**Published:** 2020-05-20

**Authors:** Siddharth Kawadiya, Claire Welling, Sonia Grego, Marc A. Deshusses

**Affiliations:** 1 Department of Civil and Environmental Engineering, Box 90287, Duke University, Durham, NC 27708, USA; siddharth.kawadiya@duke.edu; 2 Duke University Center for WaSH-AID and Department of Electrical and Computer Engineering, Durham, NC 27701, USA; claire.welling@duke.edu (C.W.); sonia.grego@duke.edu (S.G.); 3 Duke Global Health Institute, Box 90287, Duke University, Durham, NC 27708, USA

**Keywords:** odor, malodor, electronic nose, low-cost sensors

## Abstract

Technology innovation in sanitation is needed for the 4.2 billion people worldwide, lacking safely managed sanitation services. A major requirement for the adoption of these technologies is the management of malodor around toilet and treatment systems. There is an unmet need for a low-cost instrumented technology for detecting the onset of sanitation malodor and triggering corrective actions. This study combines sensory data with low-cost gas sensor data on malodor emanating from feces. The response of 10 commercial electrochemical gas sensors was collected alongside olfactometric measurements. Odor from fecal specimens at different relevant dilution as well as specimens with pleasant odors as a control were evaluated for a total of 64 responses. Several of the sensors responded positively to the fecal odor, with the formaldehyde, hydrogen sulfide, and ammonia sensors featuring the highest signal to noise ratio. A positive trend was observed between the sensors’ responses and the concentration of the odorant and with odor intensity, but no clear correspondence with dilution to threshold (D/T) values was found. Selected sensors were responsive both above and below the intensity values used as the cutoff for offensive odor, suggesting the possibility of using those sensors to differentiate odor offensiveness based just on the magnitude of their response. The specificity of the sensors suggested that discrimination between the selected non-fecal and fecal odors was possible. This study demonstrates that some of the evaluated sensors could be used to assemble a low-cost malodor warning system.

## 1. Introduction

Today, about 61% of the world’s population, or more than 4.2 billion people, lack access to safely managed sanitation services [1]. Poor sanitation, the spread of fecal pathogens, and associated diarrheal diseases are responsible for increased morbidity and mortality of millions of people. The lack of safely managed sanitation services is also disproportionally affecting women, girls, and elderly people [1,2]. In order to decrease the burden of disease related to poor sanitation, the WHO conceptualized the Sustainable Development Goal (SDG) 6.2 [2], which aims to provide sanitation for everyone by 2030. This SDG has motivated many researchers, NGOs, and governments to place efforts in the development of new sanitation and fecal sludge management (FSM) technologies and to develop major campaigns for increased access to sanitation. One such effort is the Reinvent the Toilet Challenge (RTTC) by the Bill and Melinda Gates Foundation, which focuses on innovative toilets and sanitation [3]. A major risk factor in the sanitation chain, from capture to treatment and reuse of excreta, is malodor. Fecal odor is a major barrier for toilet use; we have unpublished results indicating that 36% of respondents to a survey on toilets and odors in less developed countries would choose open defecation rather than use a malodorous toilet. Nuisance caused by fecal malodors is also a major concern for the treatment of fecal sludge and resource recovery processes [4,5].

Fecal malodor is a complex mixture of sulfur and nitrogen-containing compounds and fatty acids, along with other compounds at trace levels [6,7]. Table 1 contains a list of the major fecal odorants and their typical concentrations in pit latrines. For many of these compounds, the odor threshold, defined as the lowest concentration at which the odorous compound can be perceived by 50% of a panel of experts, is extremely low (Table 1).

Olfactometry refers to the measurement of odor intensities or concentrations. The most common method of determining odor concentration is by using an olfactometer to determine the dilution to threshold (D/T) of a sample. The D/T of an air sample refers to the number of times the sample has to be diluted before it can not be detected by the assessor. A higher D/T corresponds to a higher odor concentration in the original sample [9]. There are many challenges in describing and quantifying complex odors. One such challenge is the fact that the odor concentrations (reported in D/T) [9], odor intensities, or hedonic tones [10] cannot be singularly linked to the concentrations of odorous compounds [11,12]. This is because odor perception by humans varies based on each individual sensitivities. In addition, congruent (i.e., additive) and antagonistic (i.e., canceling) effects between odorants will alter human perception [13]. Even so, there is a great need to develop compact electronic and real-time methods to detect and quantify odors, particularly for situations where continuous odor monitoring is required, as in the case of novel toilets and sanitation technologies. Such a method would be able to provide remote monitoring and allow early alerts for system malfunction. 

Electronic noses or e-noses are devices that use sensors often coupled with a pattern recognition system to detect and sometimes quantify or classify odors [14,15]. E-noses have been around for a few decades; they are highly customizable since the sensors comprising the e-nose can be selected based on target gases [16]. However, thus far, e-noses have had limited use in industrial settings for a variety of reasons, including low sensitivity, low accuracy, and low reliability [14,15]. Probably, the most successful applications have been in the food industry for quality control and spoilage detection [17]. Still, a daunting challenge in all e-nose applications is the correlation of sensor data with sensory observations, namely odor concentration, odor intensity, and hedonic tone. 

A few investigators have attempted to deploy e-noses in sanitation-related applications. Lorwongtragool et al. [18] used metal oxide sensors (MOS) combined with principal component analysis (PCA) to detect malodorous compounds in swine buildings and relate the sensor responses to pleasant and unpleasant odors. They showed that as more time elapsed after cleaning the swine barns, the sensors’ responses increased, showing an increase in the odor. PCA was able to differentiate between a pleasant odor (after cleaning) and an unpleasant odor (a few hours after cleaning), with PC1 having a 94% variance. Teo et al. [19] utilized tin-oxide gas sensors coupled with a multi-layer perceptron back propagation algorithm to analyze human odors like urine, feces, garlic, sweat, etc. for search and rescue operations. They found that the artificial neural network (ANN) could not correctly identify the components of mixed odors since the response of the sensors to a mixed dose is not a sum of their responses to individual odors. Hirano et al. [20] were successfully able to use a combination of MOS sensors that reacted to odors present in urine and feces, combined with a decision tree classifier to classify bathroom activities like showering, urinating, and defecation with 93% precision. However, since olfactometry data were not collected, the sensor response cannot be used to determine whether a given activity produced odors that were offensive. The current state of research shows that while sensors are generally successful in identifying components of urine and feces, the lack of data on human perception of the smell in these applications proves to be a barrier in differentiating between an offensive and a non-offensive odor based purely on the sensor responses. 

Some attempts were made to combine e-noses with dynamic olfactometry to relate electronic signals to sensory data. Giungato et al. [21] measured odors in waste treatment plants using e-noses made of MOS and nanocomposite array sensors and found a good correlation between the sensor responses and the odor concentration found by dynamic olfactometry (R^2^ = 0.97). Additionally, Milan et al. [22] found that responses from e-noses comprised of MOS sensors [23] installed in restaurants correlated to the recorded odor perceived by nearby residents 90% of the time. These examples demonstrate the strength of combining sensory and sensor data. Possibly, this approach could be adapted in the field of FSM by choosing sensors that respond to malodorous gases present in fecal odor.

In this report, the possibility that a combination of low-cost sensors could provide an effective, real-time odor detection, and act as an early warning system for process malfunction was investigated. Ten low-cost sensors potentially sensitive to fecal and other odors were selected and assembled in a compact device for real-time odor monitoring. The responses of the multiple sensors to common fecal odors (fresh feces and thermally dried feces) and other odors at different relevant dilutions were collected alongside olfactometric measurements.

## 2. Materials and Methods

### 2.1. Gas Sensors Selection and Characteristics

Table 2 lists the sensors selected for this study; all sensors were electrochemical. The selection criteria for the sensors included high sensitivity, the probability to respond to fecal odorants, and cost. The retail prices of the sensors ranged from $60 to $140, with the exception of the CityTech sensors for mercaptan ($379) and tetrahydrothiophene ($550). Originally, MOS sensors that generally cost less than $20 (retail) each were considered, but they were not further explored because their typical lower limit of detection was in the ppm range, while the odorant levels expected were in the ppb range (Table 1). The probability of responding to fecal odorants included consideration of cross-sensitivity of some sensors to likely fecal odorants, including sensors with broad sensitivity, which could possibly serve a purpose for the desired application (e.g., tetrahydrothiophene for sulfur compounds). The Cairsense H_2_S/CH_4_S (Cairpol) was an integrated air quality monitor that was available to our research group and was used for relative humidity measurement. 

### 2.2. Sensor Assembly Device

A 26 × 16 × 9 cm diecast aluminum NEMA enclosure (Polycase, Avon, OH, USA) was used as the housing for the gas sensors. The sensors were mounted on their respective circuit boards, which were glued to the inside of the box. Ports were drilled into the box for wiring the sensors. All sensors, except those from SGX, were connected to 2 data acquisition (DAQ) interfaces (DI-1100, Dataq Instruments, Akron, OH, USA) through a breadboard, which provided the circuit and power for the sensors. The Membrapor and CityTech sensors were powered by a 12 VDC power supply. Additionally, each of the Membrapor sensors needed a 250 Ω resistor in the circuit for proper data acquisition. The SGX sensors came with independent power, USB connectors, and DAQ software. The two DAQ interfaces and the SGX sensors were connected to a laptop computer. Data collection was conducted using WinDAQ (Dataq Instruments, Akron, OH, USA) and SGX CVQ-EK3 (SGX Sensortech, Neuchatel, Switzerland) for the DAQ interface and the SGX sensors, respectively. The Cairsense air quality monitor was also placed in the enclosure and logged data, including relative humidity values that could be downloaded post-experiment using a USB connection. 

### 2.3. Odor Generation and Collection

The samples for fecal odor generation tested included dog feces, human feces (Duke IRB #: 2019-0010).), feces subjected to thermal drying, and non-fecal odor of popcorn and an odor control powder (citrus smell, Organico, Centurion, South Africa). The former samples were selected to test malodors that could be present in toilet systems or during thermal processing. They represented only a subset of possible fecal odors that could emanate from toilet usage and fecal sludge management practices. For example, the sample aging known to affect odor was not considered. The non-fecal odors were selected to assess the ability of the sensor system to distinguish these selected pleasant odors from the fecal malodors.

**Figure 1:**
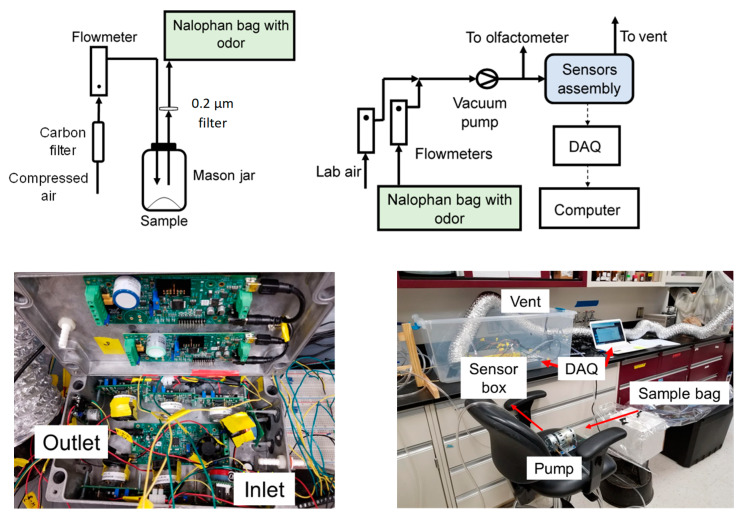
Schematic of the experimental setup for odor generation and collection (**top left**), and odor analysis (**top right**), and pictures of the sensor box (**bottom left**) and of the overall test system (**bottom right**).

For each experiment, 150 L odorous air was collected as follows in a Nalophan sample bag. For each run with fresh feces and the odor control product, 100 g of the selected fecal matter or odorant was placed in a 450 mL Mason jar fitted with an inlet and an outlet gas port (see Figure 1, left). Compressed air was passed through a carbon filter to deodorize it and passed through the jar at a flow rate of 4 L min^−1^. The outlet port was connected to a 0.2 µm disk filter to prevent possible airborne microorganisms from the fecal sample from contaminating the odor sample bag. The sample bags were custom made with 2 m long and 40 cm wide pieces of Nalophan NA20 (Kalle USA, Gurnee, IL, USA) sealed with cable ties at both ends and fitted with gas connectors. Nalophan is odorless and inert and is widely accepted as the best material for odor sample bags. For each experiment, 150 L of odorous air was collected in single-use sample bags; the odorous air was allowed to mix and equilibrate for 10 min before analysis. 

The odorant samples included human and dog feces, feces subjected to thermal drying, popcorn, an odor control powder (citrus smell, Organico, Centurion, South Africa), and water only (as a humidity control) (see Table 3). For odor collection during feces drying, 10 g of human feces were collected in a drying pan and dried at 70 °C in a small convection oven. Headspace air was collected, starting when the fecal material reached 70 °C and collection lasted 20 min. Air was sampled directly from over the drying pan using a vacuum pump at 8 L min^−1^ and collected in the sample bag. A 0.2 µm disk filter was used before the vacuum pump to eliminate bioaerosols.

For the assessment of the impacts of the air relative humidity, 250 mL of hot water (~75 °C) was placed in a 4 L open container inside the sealed sample bag, and air at 4 L min^−1^ was passed over it. Once the required volume of air was collected (generally 150 L), the side of the bag containing the container was sealed off to isolate the source from the air sample, and the other side was used for gas analysis. A reasonable assumption for this sample was that air was close to saturation for water vapor (~100% relative humidity) after cooling to room temperature. Actual relative humidity levels were recorded by the Cairpol sensor. 

For odor collection during popcorn odor runs, a packet of microwavable popcorn (Orville Redenbacher’s) was microwaved for two minutes. The packet of popcorn was opened and placed in the sealed Nalophan sample bag, and air at 4 L min^−1^ was passed over it. Once the required volume of air was collected, the side of the bag containing the popcorn was sealed off, and the other side was used for odor analysis. During the first run, it was observed that some of the popcorn was burnt. Table 3 provides a summary of the runs conducted.

### 2.4. Experimental Setup and Protocol for Odor Sensing Experiments

The schematic of the experimental setup is shown in Figure 1. A vacuum pump and 2 vacuum flowmeters (0–10 L min^−1^, Dwyer, Michigan City, IN, USA) were used to pump and appropriately dilute air from a bag containing a concentrated odor sample (see Section 2.3) and pass the resulting air mixture through the sensor box. One of the flowmeters was connected to the odor sample bag, while the other was left open to the ambient air to achieve the proper dilution. All tubing was food grade Tygon (5 mm ID), thus minimizing odor adsorption. A sample port was placed before the sensor box for olfactometric measurements. Although some odor might have been adsorbed by the pump and the Tygon tubing, the placement of both the sensor box and the olfactometry port after the pump meant that all sensors and the panelist were exposed to the same odor. This ensured that the comparison of sensor response and olfactometry readings was accurate. The output from the sensor box was vented. 

Each experiment was started by switching on all sensors for 90 min to allow them to reach a stable baseline. Ambient air was then pumped at a flow of 4 L min^−1^ through the sensor box for 1–2 min to flush the box and acquire a stable baseline. Next, H_2_S and NH_3_ standards (Airgas, Radnor, PA, USA) at concentrations of 1 ppm and 50 ppm, respectively, were passed through the sensor box at 2 L min^−1^ each for 9 min, followed by ambient air for 6 min. This was followed by feeding the odor sample at flowrates of 3.5, 2.5, 2.0, 1.5, 1.0, and 0.5 L min^−1^, with ambient air being used as makeup gas to keep the total airflow rate at 3.5 L min^−1^. Thereafter, these conditions were referred to as 100%, 71%, 57%, 43%, 29%, and 14% strength, respectively. At each dilution step, the odorous air was pumped for 9 min, followed by 6 min of ambient air to allow the sensors to reach baseline again. Olfactometry (dilution to threshold) and odor intensity measurements were conducted at each step while the odorous air was passed through the sensor box (see Section 2.5). Thus, odor samples for intensity measurements were not randomized.

### 2.5. Data Analysis

For each exposure to odorous air, a signal to noise (S/N) ratio was determined. The signal was taken as the amplitude between the baseline signal output of the sensor and the signal response when the odorant was introduced, and the noise was the standard deviation of the baseline. The ratio of these 2 indicated whether the signal of the sensor was responsive enough to detect a malodor. For each experiment, the exact time was recorded when the sensors were exposed to an odorant. Using the exposure times and the raw data of each sensor, a custom Python code was developed to evaluate all S/N [24]. The steady-state odorant signal reported herein was the average of the 2 min preceding the local maximum closest to the recorded turning off time during an odorant exposure. The baseline for each odorant exposure was the average of the 30 s preceding the local minimum closest to the recorded turning on time. The amplitude was the difference between signal and baseline. The noise was calculated as the standard deviation of the 30-s baseline.

### 2.6. Analytical Methods

Dynamic olfactometry was conducted using a SM100 Field Olfactometer (Scentroid, ON, Canada) modified in house to a run triangular forced-choice method similar to the European standard EN 13725, thus providing dilution-to-threshold (D/T) [6]. In short, the in-house modification included an Arduino Uno controller (Arduino, Somerville, MA, USA), 3 solenoid valves (Grainger, Lake Forest, IL, USA), and a custom code running the method and collecting the panelist inputs. For all olfactometric analyses reported herein, 1 single trained panelist was used. This was a deliberate choice motivated by the observation that within one experiment for 1 odorant, there was relatively little spread between the D/T of the undiluted sample and the D/T of the most diluted sample, i.e., the specimen had a strong odor. This greatly decreased the statistical significance of the results obtained by a multi-person panel, because the variance of D/T between panelists would have been greater than the D/T difference within one experiment. Although not ideal, this could be avoided by using a single trained panelist who was tested with a reference odor, according to EN 13725 [6]. The odor analysis with the modified olfactometer was conducted as follows. For each sample, the panelist selected a starting dilution and was presented successively 3 air samples (A, B, and C) to sniff. Out of the 3 air samples, 2 were blanks and 1 contained the odor sample. The panelist had to make a selection for which of A, B, or C was the odor sample, and the program advanced to the next level in a geometric fashion, to less diluted samples. This triangular forced-choice method was similar to EN 13725. The analysis was stopped when the panelist was able to successfully identify the odor sample for 2 consecutive levels. The dilution to threshold (D/T) was the geometric mean of the dilution at the 1^st^ correct identification and of the previous dilution [10]. For example, if the panelist successfully identified the odor sample at dilution levels 9 (corresponding to a D/T of 94.1), and 10 (D/T = 53.4), the D/T of the sample would be:

D/T_sample_ = Geometric mean (D/T_dilution_level_8_ and D/T_dilution_level_9_) = Geometric mean (207.6, 94.1) = 139.8

The actual dilutions of the Scentroid instrument were verified bimonthly using a calibrated H2S standard and a Jerome 631-X Hydrogen Sulfide Analyzer (Arizona Instruments, Chandler, AZ, USA).

In addition, odor intensities were recorded for all air samples passed through the sensor box. A 6-point scale was selected according to levels described in Table 4.

## 3. Results

### 3.1. Electrochemical Sensors Response

Before installation in the sensor box, all H_2_S and NH_3_ sensors were tested with standard concentrations of the relevant target gas to check the accuracy of their factory calibrations. Prior to each run with electrochemical sensors, all sensors were exposed to H_2_S and NH_3_ standards as a positive control for H_2_S and NH_3_ sensors. Initially, this also served to determine potential cross sensitivities of other sensors to these two gases commonly present in fecal odor. As expected, all H_2_S and NH_3_ sensors were responsive when challenged with their respective target gases, and responses were consistent with expected values based on sensor sensitivities and calibrations. The Membrapor and SGX H_2_S sensors had a similar response patterns, but the magnitude was different because of their distinct sensitivities. Additionally, the Membrapor formaldehyde sensor responded to H_2_S with an output greater than that of the Membrapor H_2_S and SGX H_2_S sensors. On average, S/N response of the formaldehyde sensor to H_2_S was 1.3 times higher than that of the Membrapor H_2_S sensor and 5 times higher than the SGX H_2_S sensor. This was due to the sensor’s high cross sensitivity to S-containing compounds (stated in the sensor specifications) and its high rated sensitivity. No other sensor was cross-sensitive to either H_2_S or NH_3_.

**Figure 2:**
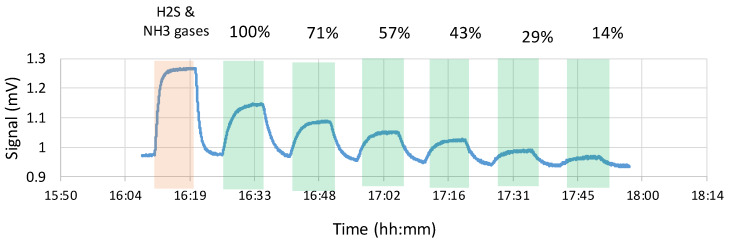
Typical dose response vs. time for Membrapor formaldehyde sensor when exposed to air containing fecal odor. The green bands indicate exposure to malodor. The values above the graph indicate the concentration with respect to the original odor sample (i.e., 100% is undiluted, 71% means 71% odor sample, and 29% odorless air).

Figure 2 reports the results of one sensor (the Membrapor for formaldehyde) during a typical dose-response experiment. After the initial positive control exposure to 1 ppm_v_ H_2_S, the sensor was exposed repeatedly to increasing dilutions of air containing the fecal odor. As shown in the figure, the sensor response decreased as the fecal odor sample dilution increased. A slight drift of the baseline can be observed, but because of our methodology, it did not affect the results (see Methods section). Typically, this sensor’s apparent response time was 2.5 min to reach 63% of the steady-state value (t63) and 4.5 min to reach 90% of the steady-state value (t90). Other sensors have t63 and t90 values ranging from 1.3 to 3.3 min, and 2.7 to 5.4 min, respectively (see Table S1, in SI). Actual response times were much faster since the time constant for concentration change in the enclosure was 1.1 and 2.5 min, for 63% and 90% of a step change, respectively, assuming ideally mixed gas phase in the enclosure. Examination of Figure 2 reveals that even at the highest dilution, the sensor had a good response to the fecal odor. The response was about 0.027 mV above the baseline, and the S/N ratio was 12.7.

### 3.2. Olfactometry Results

**Figure 3:**
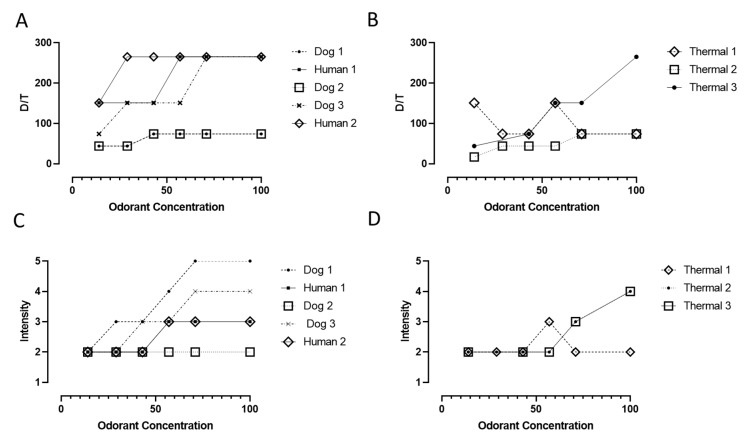
Dilution to threshold (D/T) (**A** and **B** panels) and odor intensity (**C** and **D** panels) of fecal odor samples tested with the low-cost sensors as a function of their concentration with respect to the original odor sample (i.e., 100% is undiluted). See the Methods section for details.

The olfactometric characteristics of human and dog fecal malodor air samples that were tested are shown in Figure 3. Typically, the D/T values ranged from low values of 44 to 265. Again, D/T or dilution to threshold represents the number of times the air sample needed to be diluted so that the odor was not detectable by the panelist. Measurements with respect to concentration indicated a general upward trend with some exceptions (e.g., thermal test #1), generally not spanning more than 2 or 3 dilution steps on the olfactometer, making it more difficult to obtain fine discrimination between different levels. As explained in the Methods section, this narrow spread was the motivation to use a single panelist to increase the statistical significance of the results by eliminating the panelist to panelist variability. As a result, measurements were generally repeatable. 

D/T levels as measured (44–265) were low compared to malodorous air in e.g., the waste processing industry where D/T exceeding 1000 are often encountered. As will be discussed later, all undiluted fecal odor samples were characterized as being very strong and posed significant risk of nuisance. In Figure 3A, two different specimens, one dog and one human specimen overlapped exactly.

D/T values may not be the best descriptors for assessing odor nuisance [26], mostly because the measurement is primarily sub-threshold, i.e., it does not expose the odor panelist to intense odor. Invernizzi et al. [26] proposed the use of an odor nuisance index comprising of five factors: Frequency, intensity, duration, offensiveness, and location instead of only using only the D/T values to better determine the impact of malodors. Thus, it can be argued that odor intensity is a better indicator of potential nuisance, in part because it is a supra-threshold assessment and because it is more directly connected to the magnitude of the odor perception. When using a 0–5 point scale (Table 1), values of 3 and above represent the tipping point for nuisance. Examination of Figure 3C,D shows that of the odor samples tested, most had values spanning above and below that value with dilution, thus providing an opportunity to test sensors with a range of malodor intensities. The feces samples subjected to thermal drying resulted in less offensive odors (with the exception of Thermal sample #3). This could be attributed to the odor collection method.

These data in Figure 3A,B were analyzed using Steven’s law to determine the effect of changing odorant concentration on the Dilution-to-Threshold:

C = m × [odorant]^n^

where C is the odor expressed in OU/m^3^, [odorant] is the concentration of odorant (here the percentage of the full strength was used) and m and n are constants that vary with the composition of the mixture. The values of n were generally higher (0.56–0.89) for feces subjected to thermal drying than those for the other fecal samples (0.26–0.63), showing that D/T values for feces subjected to thermal drying were more likely to be impacted by changes in odorant concentration. It is reasonable to assume that most volatile compounds, which have the lowest detection thresholds, are removed during the thermal process. As the concentration of the remaining compounds decreases upon dilution with ambient air, the corresponding D/T values decrease. For the other fecal samples, the presence of compounds like H_2_S with low detection thresholds probably dominates the overall odor profile of the sample. Hence, even a large change in the H_2_S concentration might not significantly reduce the D/T value.

### 3.3. Relationship of Sensor Responses and Olfactometry 

**Figure 4:**
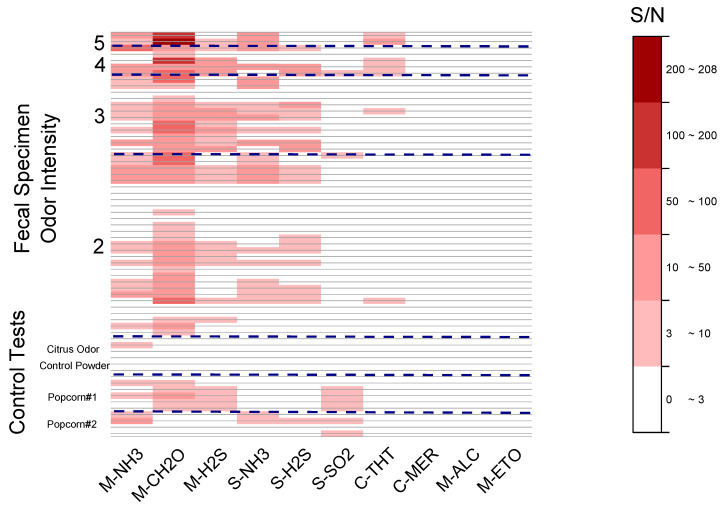
Heatmap showing the signal to noise (S/N) ratios to 64 odorant exposures for the 10 sensors tested. The horizontal dashed lines separate the different odor intensity levels.

Figure 4 is a heatmap of the signal to noise (S/N) ratios calculated for 47 sensor exposures to fecal odor and 17 exposures to non-fecal odors. The 47 fecal exposures were characterized and grouped using their intensity. Non-fecal odors were grouped by odorant type (two popcorn experiments and one commercially available odor control product). During the experiment, a humidity change, typically 7–10% RH increase was measured in the sensor enclosure during exposure to fecal odors. To allow comparison across exposures and samples, the signal response used to calculate the S/N in the heatmap was corrected for humidity as recorded by the humidity sensor located in the enclosure. A recognized correction method was used that relied on the calibration of the sensor to RH changes, fitting the sensor response to RH, and subtracting the humidity effect from the amplitude [27,28].

Figure 4 provides a snapshot of the 640 sensor responses for the 10 sensors investigated in this study. Three sensors, mercaptan, alcohol, and ethylene oxide, did not respond in any of the tests, while the SO_2_ sensor was only responsive to some non-fecal odors. The highest S/N was measured for CH_2_O, while both ammonia sensors were also responsive to fecal odors, particularly for intensities of 3 and higher. The Membrapor H_2_S sensor was more responsive to fecal malodor than the SGX. Figure 4 also illustrates that the sensor responses were generally higher with higher odor intensity.

The specificity of the sensor to fecal malodor was tested by exposing them to confounding non-fecal odor (popcorn, odor control product with perfume). There was no significant response to the perfumed odor control product (with the exception of one point), however, the response to popcorn odors was high for the most responsive sensors (Figure 4). The high response to a popcorn test was attributed to the popcorn being burnt, as suggested by responses that were greater in a run during which a burnt smell was very noticeable. Hirano et al. [29] reported the same increase in response of NH_3_ and H_2_S sensors towards popcorn as it started burning. Interestingly, the SO_2_ sensor, which did not respond to most fecal malodor samples, did respond to the popcorn smell. This may prove useful in a future device, to enhance its specificity and ability to discriminate non-fecal and fecal odors. Advanced data analysis such as principal component analysis (PCA) or pattern recognition may be needed to improve the reliability of odor discrimination. We reported a limited PCA analysis in a conference proceeding Zhou et al. [24] illustrating the possibilities of such analysis.

**Figure 5:**
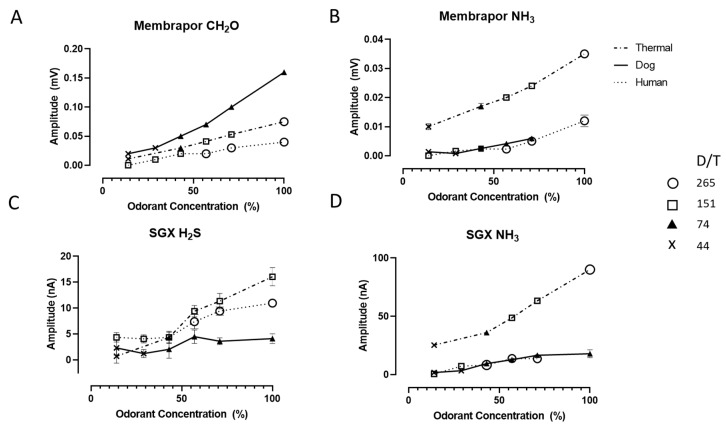
Dose response to three fecal malodor specimens by four sensors, two Membrapor (CH_2_O and NH_3_, **A**,**B**), and two SGX sensors (**C**,**D**). The dose (%) represents the concentration with respect to the original odor sample. The olfactometry data of each sample (reported as dilution to threshold, or D/T) is indicated by the marker type. Error bars represent the standard deviation.

In Figure 5, the responses of 4 of the 10 sensors exposed to different specimens of fecal malodor were reported. In each case, a positive trend can be observed between the sensors’ responses and the concentration of the odor (as reported with respect to the original malodor sample). The formaldehyde sensor (Figure 5A) exhibited a clear linear response (R^2^ > 0.98) with the concentration of each given odor sample. The other three sensors had less linear responses, and at low odor concentration, some data scattering and low signals could be observed (see SI for supplementary figures of the remaining sensors (Figure S1) and Table S2 with R^2^ values). 

Examination of the symbols in Figure 5 (representing the different D/T values) revealed that within a given odor sample, there was generally a concurrent increase in the sensor signal obtained with an increase of the D/T value (e.g., Membrapor NH_3_ signals for feces subjected to thermal drying). However, there was no correspondence between D/T and sensor signals across odor samples. For example, the response of the Membrapor CH_2_O sensor to undiluted dog feces odor was about twice that of the feces subjected to thermal drying, despite having a much lower odor threshold (53 D/T vs. 208 D/T). This is likely because of odorants in the air samples that do not elicit any sensor response. 

**Figure 6:**
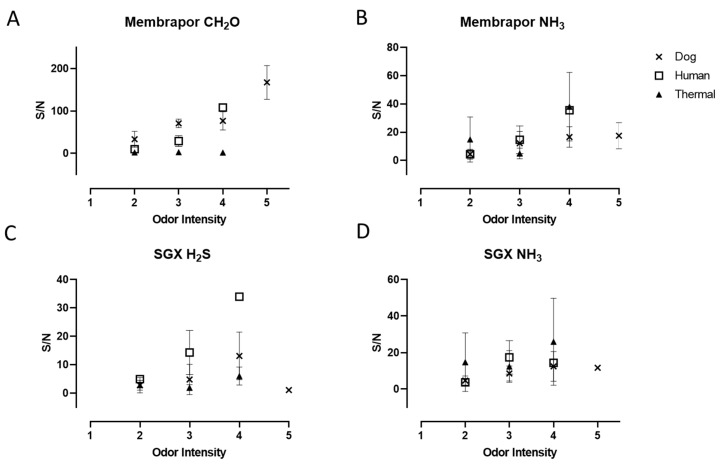
S/N ratios for three malodors specimen by four sensors (same as Figure 5) as a function of the odor intensity. Error bars represent the standard deviation.

The quantitative relationship between selected sensor responses and the odor intensity is reported in Figure 6. As mentioned before, an intensity of 3 was recognized as the cutoff for offensive odors. As shown in Figure 6, the selected sensors were responsive at levels both above and below this critical threshold for all types of fecal odor sources. This indicates that those sensors could conceivably be used to differentiate between an inoffensive and an offensive odor based on just their signals or a combination of their signals, thus suggesting that they could possibly replace olfactometry, especially in those cases where continuous monitoring is desirable. 

## 4. Conclusions

Several of the 10 electrochemical gas sensors evaluated here responded to fecal malodors including ammonia, H_2_S, and formaldehyde sensors (the latter as a result of H2S cross-sensitivity). A direct correlation was observed between some of the sensors’ responses and the concentration of the odorant. Sensors for the same target gas and from different vendors had responses of different magnitude. The response time was generally less than 2 min. The olfactometric evaluation of fecal malodors revealed that odor intensity rather than D/T value was found a better indicator of odor nuisance. Selected sensors were responsive both above and below the intensity values found to be the threshold for offensive odor, suggesting that those sensor responses could be used to differentiate odor offensiveness in practical application. Furthermore, sensors responding to fecal malodor did not respond to a perfume odor control, and when they responded to a food odor, an additional sensor (SO_2_) also responded, suggesting that a combination of multiple sensors would provide specificity. The Membrapor formaldehyde sensor, with either the SGX or Membrapor NH_3_ sensor and the SGX SO_2_ sensor, is a promising sensor combination for our application, though other sensors may work too. Overall, these results show that these low-cost sensors are well-suited to the development of malodor warning systems for sanitation applications. Future studies are needed to integrate the sensor response with a pattern recognition algorithm and to downselect the sensors and integrate them with microelectronics to optimize performance for an alert of the onset of fecal malodor.

## Figures and Tables

**Table 1:** Major odorous compounds found in toilets and latrines, their typical smell, and commonly reported odor threshold. The latter often vary by many orders of magnitude, thus average commonly reported values were selected.

Compound	Concentration in Odorous Latrines (ppb_v_)	Odor Character	Odor Threshold (ppb_v_)
Hydrogen sulfide	25–55 [7,8]	Rotten egg	0.5–2
Ammonia	50–60 [8]	Sharp, pungent	3000–20,000
Butyric acid	36 [7]	Rancid, vomit	10–500
Methyl mercaptan	2–15 [8]	Rotten egg, fermented cabbage	1–20
Indole	0.31 [7]	Fecal, musty	5–20
p-cresol	1.2 [7]	Animal barn, medicinal	0.05–9
Acetic acid	3–10 [8]	Sour, vinegar	400–1000
Propionaldehyde	10 [8]	Sweet, ester	50–200
Trimethylamine	10–100 [7]	Stale urine	50–200

**Table 2:** List of sensors and their characteristics (all sensors are electrochemical).

Brand	Sensor	Retail Price (USD)	Gas	LDL ^1^ (ppm_v_)	Range (ppm_v_)	Sensitivity (nA/ppm_v_)
SGX	SGX-7H2S	$45	H_2_S	<0.1	0–50	1700 ± 400
Membrapor	H2S/C-10	$87	H_2_S	0.003	0–10	4500 ± 1000
SGX	EC4-20-SO2	$98	SO_2_	0.1	0–20	400–600
SGX	SGX-4NH3	$88	NH_3_	1	0–100	100 ± 30
Membrapor	NH3/CR-200	$140	NH_3_	0.06	0–200	90 ± 18
Membrapor	Alc/C-100	$130	MeOH/EtOH	0.03	0–100	1600 ± 600
Membrapor	CH2O/C-10	$105	CH_2_O	0.003	0–10	4600 ± 1200
Membrapor	ETO/C-20	$105	Ethylene Oxide	0.006	0–20	2500 ± 600
CityTech	Sensoric TBM 2E 50	$379	Mercaptan	<0.14	0–14	40–100 ^2^
CityTech	Sensoric THT 3E 50	$554	Tetrahydrothiophene	<0.42	0–14	500 ± 180

^1^ LDL = Lower detection limit. ^2^ for Ethyl-SH.

**Table 3:** Summary of the runs conducted.

Odorant Sample	Number of Runs
Dog feces	3
Human feces	2
Thermally dried feces	3
Popcorn	2
Citrus odor control product	2
Water vapor (effect of relative humidity)	1

**Table 4:** Odor intensity scale, modified from Turk et al. (1980) [25].

Odor Intensity Score	Description	Details
0	No odor	-
1	Very faint	Can detect presence of an odor, but unable to assign a character to the odor
2	Faint	Can detect an odor and assign character to the odor after some thought
3	Easily detectable	Can detect odor and assign character to the odor instantaneously
4	Strong	Unpleasant odor. Can withstand it, but would prefer to move away from the odor source
5	Very strong	Unacceptable odor. Would move away from the odor source as soon as possible

## Supplementary Materials

The following are available online at https://www.mdpi.com/1424-8220/20/10/2888/s1, Table S1. Response time of the system to reach 63% (T63) and 90% (T90) of the steady-state value. Table S2. R^2^ values for the dose-response of sensors to the three fecal odorants. Figure S1. Dose-response to three fecal malodor specimens by the sensors not shown in the main manuscript.
