# Supplementary material for: Fecal Malodor Detection Using Low-Cost Electrochemical Sensors
Source: Sensors (Basel). 2020 May 20;20(10):2888. doi: 10.3390/s20102888 (PMC7287618; doi:10.3390/s20102888)
Supplement: Supplementary file 1 [file sensors-20-02888-s001.pdf]

sensors

MDPI

Supplementary Materials

Fecal Malodor Detection Using Low-Cost Electrochemical Sensors

Siddharth Kawadiya [1], Claire Welling [2], Sonia Grego [2] and Marc A. Deshusses [1,3,*]

[1] Department of Civil and Environmental Engineering, Box 90287, Duke University, Durham, NC 27708, USA; siddharth.kawadiya@duke.edu

[2] Duke University Center for WaSH-AID and Department of Electrical and Computer Engineering, Durham, NC 27701, USA. claire.welling@duke.edu (C.W.); sonia.grego@duke.edu (S.G.)

[3] Duke Global Health Institute, Box 90287, Duke University, Durham, NC 27708, USA

* Correspondence:marc.deshusses@duke.edu; Tel.: +1-919-660-5480

Received: 24 April 2020; Accepted: 17 May 2020; Published: 20 May 2020

Supplementary Materials

Table S1 reports the response time T63 and T90 of the 7 sensors that responded to malodor as in Figure 5. Note the sensor enclosure had a total volume of 3.8 L volume (without gas sensors and boards) and malodor air flow was set to 3.5 L/min (see Methods for details), thus resulting in a T63 and T90 of 1.1 and 2.5 min, respectively for the enclosure alone, if assuming the enclosure is behaving as ideally mixed.

Table S1. The response time of the system to reach 63% of the steady state value (T63) and 90% (T90) reported as average of at least three measurement.

Brand	Sensor	Gas	T63 (min)	T90 (min)
Membrapor	NH3/CR-200	NH_3	1.3	3.5
Membrapor	H2S/C-10	H_2S	1.6	2.7
SGX	SGX-7H2S	H_2S	1.8	3.0
SGX	SGX-4NH3	NH_3	2.2	4.2
Membrapor	CH2O/C-10	CH_2O	2.5	4.5
SGX	EC4-20-SO2	SO_2	2.5	3.2
CityCell	Sensoric THT 3E 50	THT	3.3	5.4
Membrapor	NH3/CR-200	NH_3	1.3	3.5
Membrapor	H2S/C-10	H_2S	1.6	2.7
SGX	SGX-7H2S	H_2S	1.8	3.0

Table S2. Correlation coefficient squared (R^2) values for the dose response of sensors to the three fecal odorants. Values shown are for amplitude vs odorant concentration plots.

Sample	M-NH_3	M-CH_2O	M-H_2S	S-NH_3	S-H_2S	S-SO_2	C-THT	C-Mer	M-ETO	M-Alc
Dog	0.872	0.983	0.864	0.904	0.557	0.028	0.911	0.299	0.001	0.701
Thermal	0.986	0.993	0.984	0.960	0.976	0.071	0.938	0.984	0.210	0.001
Human	0.865	0.984	0.996	0.913	0.888	0.459	0.923	0.901	0.0378	0.583

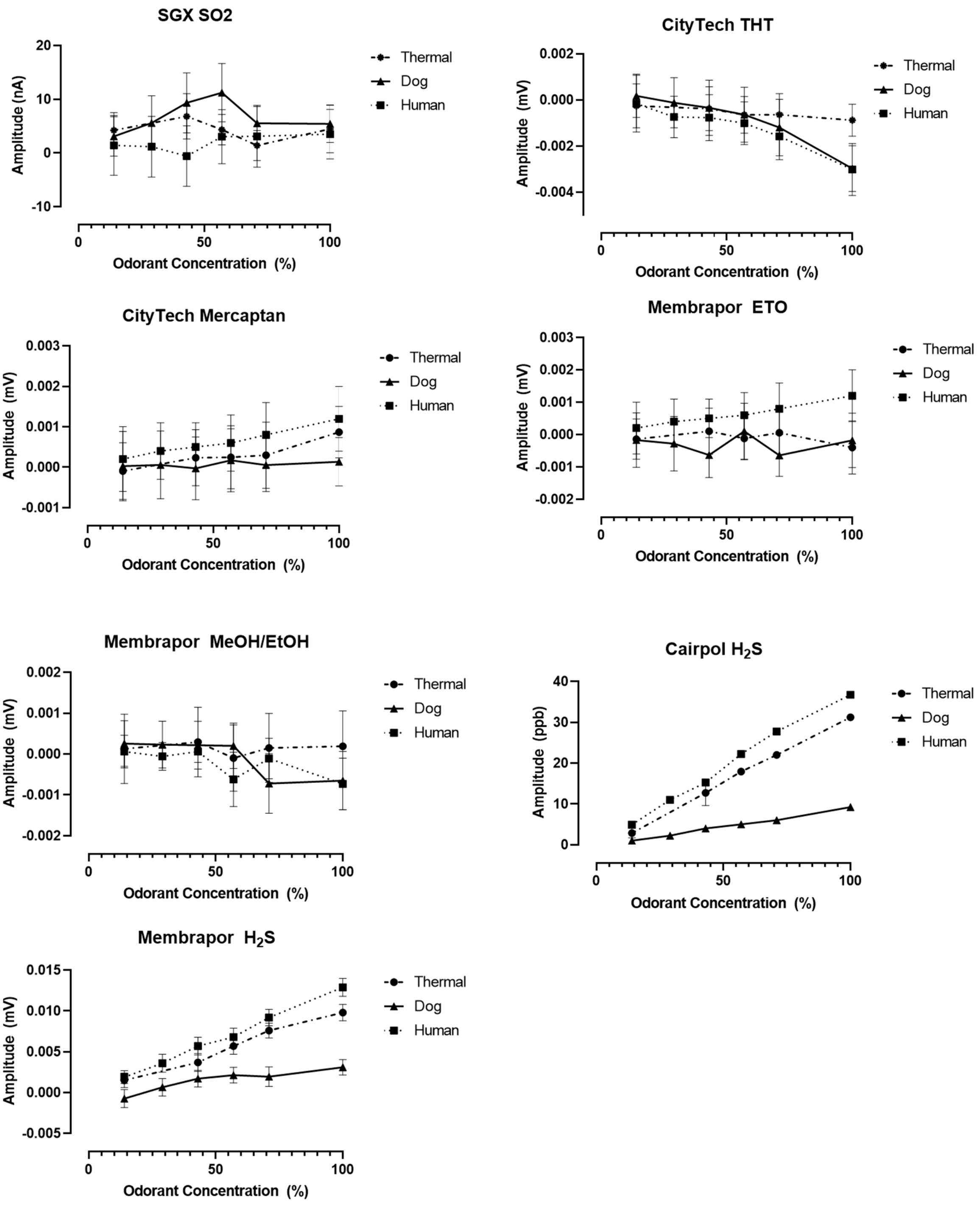

Figure S1. Dose response to three fecal malodor specimens by the sensors not shown in Figure 5 in the main manuscript. The dose (%) represents the concentration with respect to the original odor sample. Error bars represent the standard deviation. See text accompanying Figure 5 for details.

© 2020 by the authors. Licensee MDPI, Basel, Switzerland. This article is an open access article distributed under the terms and conditions of the Creative Commons Attribution (CC BY) license (http://creativecommons.org/licenses/by/4.0/).
